# Evaluation of the Effect of CD70 Co-Expression on CD8 T Cell Response in Protein-Prime MVA-Boost Vaccination in Mice

**DOI:** 10.3390/vaccines11020245

**Published:** 2023-01-21

**Authors:** Ann-Sophie Stephan, Anna D. Kosinska, Martin Mück-Häusl, Andreas Muschaweckh, Clemens Jäger, Natalie Röder, Mathias Heikenwälder, Claudia Dembek, Ulrike Protzer

**Affiliations:** 1 Institute of Virology, Technical University of Munich, Helmholtz Zentrum München, 81675 Munich, Germany; 2 German Center for Infection Research (DZIF), Partner Site Munich, 81675 Munich, Germany; 3 Institute for Experimental Neuroimmunology, Technical University of Munich School of Medicine, 81675 Munich, Germany; 4 Division of Chronic Inflammation and Cancer, German Cancer Research Center (DKFZ) Heidelberg, 69120 Heidelberg, Germany

**Keywords:** hepatitis B virus, vaccines, immune therapy, CD70

## Abstract

Here, we investigate the potential of CD70 co-expression during viral vector boost vaccination to improve an antigen-specific T cell response. To determine the chance of activating antigen-specific T cells by CD70, we used the HBV core antigen as a model antigen in a heterologous protein-prime, Modified Vaccinia virus Ankara (MVA) boost vaccination scheme. Both the HBV core and a CD70 expression cassette were co-expressed upon delivery by an MVA vector under the same promoter linked by a P2A site. To compare immunogenicity with and without CD70 co-expression, HBV-naïve, C57BL/6 (wt) mice and HBV-transgenic mice were prime-vaccinated using recombinant HBV core antigen followed by the MVA vector boost. Co-expression of CD70 increased the number of vaccine-induced HBV core-specific CD8 T cells by >2-fold and improved their effector functions in HBV-naïve mice. In vaccinated HBV1.3tg mice, the number and functionality of HBV core-specific CD8 T cells was slightly increased upon CD70 co-expression in low-viremic, but not in high-viremic animals. CD70 co-expression did not impact liver damage as indicated by ALT levels in the serum, but increased the number of vaccine-induced, proliferative T cell clusters in the liver. Overall, this study indicates that orchestrated co-expression of CD70 and a vaccine antigen may be an interesting and safe means of enhancing antigen-specific CD8 T cell responses using vector-based vaccines, although in our study it was not sufficient to break immune tolerance.

## 1. Introduction

Persistent viral infections and malignant diseases threaten millions of people worldwide and impair quality of life, cause deadly complications and lead to high costs in health care. Therapeutic vaccination offers a promising immunotherapeutic treatment option for both viral infections and malignant diseases. It has the potential to elicit an effective immune response when a suitable antigen has been defined. Chronic hepatitis B is an example of such a disease caused by persistent Hepatitis B virus (HBV) infection. The number of chronically infected individuals is high, with almost 300 million HBV carriers worldwide at high risk to develop liver cirrhosis or liver cancer causing more than 880.0000 deaths per year [1,2]. The current therapeutic options comprise treatments with nucleos(t)ide analogues or interferon alpha. However, both are rarely curative and require long-term drug administration, which is cost-intensive and prone to cause side effects, respectively [3,4,5,6].

In patients with self-limiting HBV infection, the elimination or final control of the virus is associated with strong and polyclonal CD4 and CD8 T cell responses [7,8,9,10,11]. In persistent chronic HBV infection however, HBV-specific T cells are only detected at a very low frequency and—at least partially—in an immunotolerant state, described as T cell exhaustion, resulting in a failure to eradicate the virus [12,13,14,15,16,17,18].

Therapeutic vaccination aims at empowering the immune system and restoring the functions that control the virus. It has been applied as a potential approach for the treatment of chronic hepatitis B (reviewed in [19,20,21,22]). These studies, however, have shown that therapeutic vaccination is challenging and requires well-formulated antigens, delivery systems and most likely a combination with either antiviral treatment, immune stimulators or a simultaneous blocking of inhibitory molecules [21,23,24].

We have recently developed a therapeutic heterologous prime-boost vaccination regimen, termed *TherVacB*. Based on a prime vaccination with particulate HBV antigens followed by boost vaccination with a recombinant modified vaccinia virus Ankara (MVA) expressing HBV antigens, *TherVacB* stimulates a strong anti-HBV immunity [25]. The approach elicits both anti-HBs seroconversion and strong HBV surface- and HBV core-specific CD4 and CD8 T cell immunity [25]. We have demonstrated that *TherVacB* is capable of breaking HBV-specific immune tolerance in HBV transgenic mice with low to intermediate HBV antigen concentrations, but at high HBV antigen concentrations the therapeutic vaccine failed to induce HBV specific CD8 T cell immunity [25]. Thus, the *TherVacB* strategy was successfully combined with additional treatments before, during and after vaccination in preclinical studies: namely siRNA mediated knockdown of virus antigen expression prior to vaccination [26], knockdown of PD-L1 expression during vaccination [27] and post-treatment with CpG [28]. The therapeutic effect could be significantly increased in preclinical mouse models with these treatments—even in animals with a high antigen load—up to a permanent elimination of the virus in the AAV-HBV mouse model [26].

The CD70 molecule is a surface antigen that is physiologically expressed mainly on activated mature dendritic cells (antigen presenting cells). Its ligand, the CD27 molecule, is constitutively expressed on the surfaces of resting and naive T cells. Ligation of CD70 on antigen presenting cells with CD27 on T cells promotes T cell activation [29] and survival of antigen-specific activated T cells [30,31,32,33], and is tightly regulated to prevent overexpression and excessive lymphocyte activation. CD70 co-stimulation induces proliferation of T cells and contributes to the activation of their effector function such as the increase in cytotoxic CD8+ T cell activity [32,34,35,36,37,38,39]. Further, it influences CD4+ T cell subset differentiation [36,40] and contributes to the formation of the effector and memory T cell pool [39,41]. Recent data have even shown that CD70 expressed on regulatory T cells inhibits their suppressor function and provides co-stimulation to conventional T cells [42]. All in all, CD70 is an interesting candidate to flip the switch from T cell tolerance to immunity and is therefore often the subject of investigation in immunotherapeutic approaches against cancer and chronic viral infections. However, CD70 co-stimulation in immunotherapy bears the potential risk of the over-activation or unspecific stimulation of immune cells and, therefore, promoting autoimmunity [43,44].

In order to investigate the role of CD70 co-expression in vaccine-induced CD8 T cell immunity we chose the HBV core protein as a model antigen, a strong inducer of HBV-specific CD8 T cell response. In the current study we omitted the simultaneous expression of the HBV surface protein—the most effective protective HBV vaccine candidate—in order to firstly exclude interference across specific responses and secondly avoid a robust antibody response with significant effects on HBV parameters (which would be the case by anti- HBs [26,45], but not by anti-HBc antibodies [28]). The use of the HBV core as a model antigen therefore allowed the scientific question of whether CD70 would increase T cell immunity to be addressed.

In this study, we investigated whether and how CD70 co-expression may improve the CD8 T cell response boosted by a viral vector vaccine. We generated an MVA-vector expressing the HBV core protein and integrated the co-stimulatory molecule CD70 under control of the same promotor. This system offers the opportunity of delivering the target antigen in direct spatial and temporal conjunction with the co-stimulatory CD70 protein, further reducing the risk of misdirected co-stimulation, which was already minimized by the upstream protein prime in our vaccination scheme. In this model, we showed that expression of and co-stimulation by CD70 can selectively increase and improve the CD8 T cell response against the viral model antigen in HBV-naive but not in immune-tolerant, HBV-transgenic mice.

## 2. Material and Methods

### 2.1. Generation and Production of MVA Vectors

The gene expression cassettes for recombinant MVAs were cloned utilizing the MVA shuttle plasmid pLW-73 [46]. The coding sequence of human CD70, which was demonstrated to bind to murine CD27 [47], was derived from cDNA from an Epstein-Barr Virus (EBV)-immortalized human B-cell line. For generation of the respective recombinant MVA (rMVA), DF-1 cells were transfected with the MVA shuttle plasmid and infected with wildtype MVA (MVAwt). After recombination, amplification of recombinant MVAs (rMVAs) was performed in DF-1 cells (Institute of Virology, Helmholtz Center Munich). Amplified rMVAs were purified via 36% sucrose cushion ultracentrifugation (13,500 rpm at 4° for 90 min, a second round was performed after resuspension). rMVAs were titrated in serial dilutions on DF-1 cells. The titers were determined by half-maximal median tissue culture infection dose (TCID_50_) of two repetitions. The rMVAs were stored at −80°. Three MVA vectors were used in this study: MVAwt, rMVA-HBc and rMVA-HBc-CD70.

### 2.2. Evaluation of Gene Expression in Cell Culture

HEK293 cells were transfected with the plasmids containing the gene expression cassette (CMV-Pr.-HBc[-P2A-CD70]-IRES-GFP) by using Lipfectamin2000 transfection reagent (Thermo Fisher Scientific, Waltham, MA USA 02451) according to the manufacturer’s instructions. After two days, cells were harvested and analyzed. The expression of CD70 and GFP was determined by FACS analysis of the transfected cells after LIVE/DEAD staining with the Near-IR Dead Cell Stain Kit (Life technologies GmbH, Invitrogen, Darmstadt, Germany) utilizing anti-CD70-PE antibody (BioLegend, San Diego, CA, USA). Expression of the HBV core protein was detected by Western Blot/SDS-Page of a lysate of transfected HEK293 cells. After transfer to nitrocellulose, the blotted bands were immunodetected with rabbit anti HBV-core (DAKO Carpinteria, CA, USA) as the primary antibody and subsequently visualized with peroxidase-labeled mouse anti-rabbit IgG antibodies.

### 2.3. Ethical Statement

Animal experiments were conducted in strict accordance with the regulations of the German Society for Laboratory Animal Science (GV-SOLAS) and the health laws of the Federation of European Laboratory Animal Science Associations (FELASA). Experiments were approved by the local Animal Care and Use Committee of Upper Bavaria (Permit No. 55.2-1-54-2532-103-12). For animal welfare reasons, clear trends in individual transgenic animals were described by observation without raising the groups to a statistically relevant size. Mice were kept in a specific pathogen-free facility under appropriate biosafety level following institutional guidelines.

### 2.4. Experimental Animals

Wild-type C57BL/6 mice (haplotype H-2^b/b^) were purchased from Charles River Laboratories (Schulzfeld, Germany). Fourteen to sixteen weeks old female and male HBV transgenic mice (StrainHBV1.3.32, HBV genotype D, subtype ayw, kindly provided by F. Chisari, The Scripps Institute, La Jolla, CA, USA [7]) were bred at the AVM Animal Facility, Helmholtz Center Munich under specific pathogen-free conditions following institutional guidelines.

### 2.5. Heterologous Protein-Prime MVA-Boost Vaccination

Mice were immunized with a particulate protein prime and subsequently boosted with a recombinant Modified Vaccinia Ankara virus (MVA) expression HBV core protein. As protein prime, we used recombinant HBV core antigen (HBcAg) expressed in *Escherichia coli* that consist of 180 or 240 subunit of the HBV core protein and spontaneously forms particles (APP Latvijas Biomedicinas, Riga, Latvia). Briefly, for the protein prime at day 0, mice were immunized subcutaneously with 30 µg of recombinant HBcAg adjuvanted with 32 µg of synthetic phosphorothioated CpG ODN 1668 and 25 µg poly[di(sodiumcarboxylatoethyl-phenoxy)phosphazene] (PCEP) dissolved in 50 µL PBS (group wt received adjuvants only). For the MVA-boost at day 21, mice received 10^8^ infectious units (IU) of MVAwt (group wt), rMVA-HBc (group HBc) or rMVA-HBc-CD70 (group HBc-CD70) by intraperitoneal injection in 250 µL PBS [25]. Mice were sacrificed at day 35 of the experiment for analysis of splenocytes +/− liver associated lymphocytes +/− liver sections + serum.

### 2.6. Isolation of Lymphocytes from the Spleen and Liver

Splenocytes were isolated as previously described [48]. Liver-associated lymphocytes (LAL) were isolated and purified by density gradient centrifugation as described in [12]. Briefly, mouse livers were perfused with pre-warmed PBS and pressed through a 100 µm nylon cell strainer (BD Falcon, Franklin Lakes, NJ, USA). After washing, cell pellets were suspended in 10 mL of a pre-warmed enzyme solution containing 1 mg/mL collagenase type IV (Worthington, Lakewood, NJ, USA) in RPMI 1640 medium containing 10% fetal bovine serum (Gibco, Thermo Fischer Scientific, Darmstadt, Germany) and digested at 37 °C for 30 min. The cell pellets were then resuspended in 40% Percoll solution (GE Healthcare, Munich, Germany), layered on 80% Percoll solution and centrifuged at 1600× *g* for 20 min without brakes for density separation, resulting in the LAL fraction concentrating between the two Percoll layers.

### 2.7. Detection of HBV Core-Specific CD8 T Cells by Multimers and Intracellular Cytokine Staining

HBV core-specific CD8 T cells were detected by staining with MHC class I multimers conjugated with HBV core-derived H-2K^b^-restricted peptide C_93-100_ (C_93_, MGLKFRQL), as described previously [28].

For intracellular cytokine staining, splenocytes and liver-associated lymphocytes (LAL) were stimulated with H-2K^b^-restricted peptides C_93_ (HBcAg (ayw), sequence: MGLKFRQL, (JPT Peptide Technologies, Berlin, Germany)) or B8R (MVA, sequence: TSYKFESV (kindly provided by Ingo Drexler, Heinrich Heine Universität Düsseldorf, Germany)] for 5h in the presence of 1 mg/mL Brefeldin A (Sigma-Aldrich, Taufkirchen, Germany). Cells were live/dead-stained with ethidium monoazidebromide (Invitrogen, Karlsruhe, Germany). Surface markers were stained with PB-conjugated anti-CD8 T cell antibody (clone 56.6-7, BD Biosciences, Heidelberg, Germany) and anti-CD4-PE (eBioscience, San Diego, USA). Intracellular cytokine staining (ICS) was performed using a Cytofix/Cytoperm Kit (BD Biosciences, Heidelberg, Germany) according to the manufacturer’s instructions with FITC anti-IFNƴ (clone XMG1.2, eBioscience), PE-Cy7 anti-TNFα (Biolegend) and APC anti-IL2 (eBioscience). Data were acquired on a CytoflexS (Beckmann Coulter) flow cytometer. Analyses were performed using FlowJo-Version9 software (Tree Star, Ashland, OR, USA).

### 2.8. Serological Analysis

Serum levels of HBeAg and anti-HBc were determined in 1:10 dilutions using AXSYM^TM^ assays (Abbott Laboratories, Abbott Park, IL, USA). Alanine aminotransferase (ALT) was measured from fresh serum with Reflovet (scil, Viernheim, Germany).

### 2.9. Immunohistochemistry

Liver tissue samples were fixed in 4% buffered formalin for 48 h and embedded in paraffin. Subsequently, 2 µm thin liver sections were prepared using a rotary microtome (HM355S, ThermoFisher Scientific, Waltham, MA, USA). Nuclei were stained with hematoxylin. The following antibodies were used to stain HBcAg, CD3 and CD3+Ki67: anti-HBcAg primary antibody (Diagnostic Biosystems, Pleasanton, CA; 1:50 dilution) and a horseradish peroxide coupled secondary antibody; anti-CD3 antibody (host: rabbit (IR503; Dako); ready to use) and anti-Ki67 antibody in a 1:200 dilution (SP6, NeoMarkers/Lab Vision Corporation). For quantification, sections were scanned and scored manually (40× magnification): in 6–10 randomly chosen areas of 0.5 mm^2^ on CD3-stained paraffin sections, cells (CD3+ and HBc+) were counted and merged for statistical analysis. For counts of T cell clusters, cell accumulations of >3 CD3+ cells and >6 CD3+ cells were counted in the whole sections and normalized to cell accumulations/mm^2^.

### 2.10. Statistical Analysis

Statistical analyses were performed using GraphPad Prism version 5 (GraphPad Software Inc., San Diego, CA, USA). Results are depicted as means ± standard deviations. Differences between groups were analyzed for statistical significance using a one-way ANOVA to compare all groups. If significant, unpaired t-tests were performed as post hoc tests (95% confidence level). *p*-values < 0.05 were considered significant.

For the calculation of the gain of functionality data, results of the ICS and C_93_ multimer stainings were used: the proportion of IFNγ+ CD8 T cells was divided by the proportion of C_93_ multimer-positive CD8 T cells for each mouse separately.

## 3. Results

### 3.1. Generation of Recombinant MVA Encoding CD70

**Figure 1:**
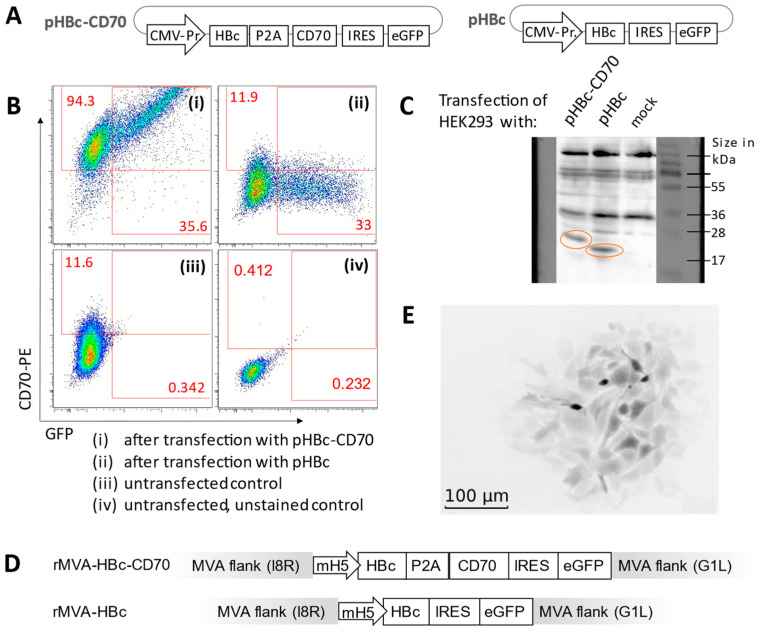
Generation of recombinant MVA vectors encoding the transgenes HBV core antigen (HBc), the reporter GFP and the co-stimulatory molecule CD70. (**A**) Schematic illustration of the plasmids pHBc-CD70 and pHBc containing the respective gene expression cassettes under control of a CMV-promoter/enhancer element. (**B**) HEK293 cells transfected with pHBc-CD70 or pHBc were stained with anti-CD70-PE (except lower right unstained control) and showed a positive correlation of CD70 and GFP co-expression (upper left). (**C**) Western blot analysis of HBV core proteins in supernatants from non-transfected (lane 3, “mock”) or from pHBc-CD70 or pHBc transfected HEK293 cells (lane 1 and 2). The circled bands indicate the HBV core protein (21 kDa) and core plus remaining aminoacids added by the P2A site (21 plus 2.1 kDa). (**D**) Gene expression cassettes of rMVA-HBc-CD70 and rMVA-HBc, which was introduced in the wildtype MVA genome by spontaneous homologous recombination with shuttle plasmids in DF-1 cell culture. The gene expression cassette is located between the essential MVA genes I8R and G1L. (**E**) Amplification of recombinant MVAs resulted in green fluorescent spots on the DF-1 cell layer detected by immunofluorescence microscopy (visible here in darker shades of grey). This allowed positive selection and removal of residual wildtype MVA and production of pure recombinant MVA. CMV-Pr.: CMV-Promoter; mH5: mH5 promoter; HBc: sequence for HBV nucleocapsid protein HBV core; P2A: self-cleaving site; IRES: internal ribosomal entry site; eGFP: sequence for enhanced fluorescent protein.

A gene expression cassette was designed encoding the HBV core antigen and the human B-cell-derived protein CD70. Simultaneous and equimolar expression of HBV core and CD70 was accomplished utilizing a P2A site [49]. The corresponding control construct was generated in analogy but without the P2A-CD70 cassette. During the production process, co-expression of GFP coupled to the HBV core gene with or without CD70 via an internal ribosomal entry site served for both recombinant vectors as reporter (Figure 1A). For both MVA vectors, transgene expression was under control of the early promoter mH5 [50]. The correct expression of the transgenes HBV core and CD70 was confirmed in HEK293 cells by Western blot and flow cytometry analyses (Figure 1B,C) resulting in the generation of MVAs rMVA-HBc-CD70 and rMVA-HBc (Figure 1D). Both vectors were amplified in DF-1 cell culture, purified and titrated using serial dilution (Figure 1E).

### 3.2. CD70 Co-Expression during Boost Vaccination Exclusively Increases the Number of Transgene-Specific CD8 T Cells in wt Mice

**Figure 2:**
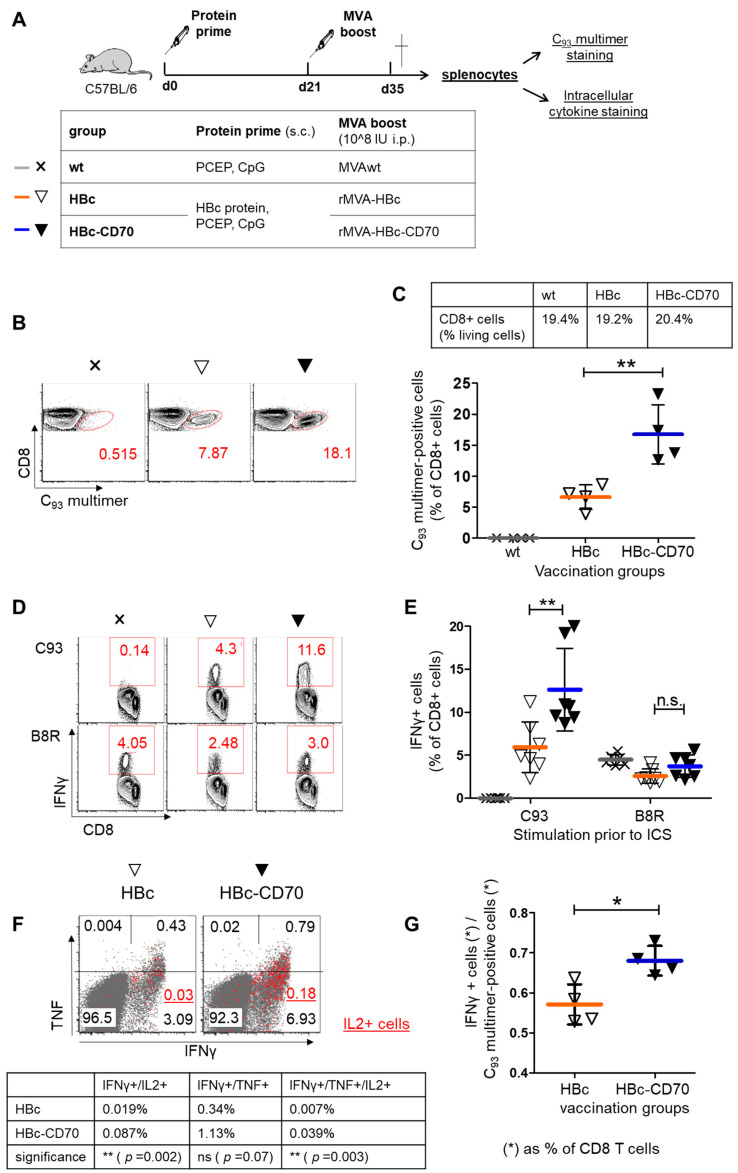
Vaccination of C57Bl/6 mice with rMVA-HBc±CD70. (**A**) C57Bl/6 mice were immunized subcutaneously with HBcAg adjuvanted with CpG and PCEP at day 0 (group wt received adjuvants only). At day 21, mice were boosted with MVAwt (group wt), rMVA-HBc (group HBc) or rMVA-HBc-CD70 (group HBc-CD70) by intraperitoneal injection. At day 35, splenocytes of mice were isolated and stained with PE-labelled C_93_ multimers to determine the amount of HBc-specific CD8 T cells (**B**) showing representative dot plots and (**C**) showing the group analysis. The table (**C**) shows the proportion of all CD8+ T cells. At day 35, splenocytes of vaccinated mice were also stimulated ex vivo with C_93_ or B8R peptides for intracellular cytokine staining (ICS) to evaluate C_93_- or B8R- specific IFNγ-, TNFα- and IL2 production of CD8 T cells (**D**) showing representative dot plots and (**E**) showing the group analysis. (**F**) Simultaneous detection of IFNγ, TNFα and IL2 production of CD8 cells upon HBc stimulation ex vivo: dot plots show one representative mouse of each group. The table shows the mean values (% of CD8+ cells) of 4 mice/group. (**G**) The gain of function of CD8 T cells after CD70 co-stimulation during vaccination: the proportion of IFNγ+ CD8 T cells upon ex vivo C_93_ stimulation was set in relation to the proportion of C_93_ multimer-positive CD8 T cells in the same animals. Proportions were calculated for each mouse separately. C_93_: HBcAg CD8 T cell epitope; B8R: MVA-derived CD8 T cell epitope. Crosses and triangles represent individual mice; horizontal lines indicate mean values; error bars indicate standard deviation. Statistics: one-way ANOVA and subsequent unpaired t-test, n.s.: not statistically significant.

Employing the well-established protein-prime MVA-boost vaccination scheme of *TherVacB* [25,26], we aimed at investigating whether CD70 co-expression during boost impacts the HBV core-specific CD8 T cell response. C57BL/6 mice were vaccinated subcutaneously with recombinant particulate HBcAg adjuvanted with a mixture of CpG and PCEP, followed by an intraperitoneal rMVA boost injection using rMVA-HBc-CD70 or control rMVA-HBc at day 21 (Figure 2A). T cell responses were analyzed 14 days after the rMVA-boost. A group of mock vaccinated C57BL/6 mice (group wt) were primed with adjuvants only and boosted with wild-type MVA (MVAwt). Splenocytes were isolated from vaccinated animals 14 days after the MVA-boost and stained with major histocompatibility complex class I multimers loaded with the C_93_ peptide MGLKFRQL.

The staining revealed that CD70 co-expression significantly increased the proportion of HBV core-specific CD8 T cells upon heterologous vaccination (from 6.6% of all splenic CD8 T cells in vaccination group rMVA-HBc to 16.8% in vaccination group rMVA-HBc-CD70; *p* = 0.0077; Figure 2B,C). In a second step we re-stimulated the T cells by loading splenocytes ex vivo with the immune-dominant C_93_ core-peptide (MGLKFRQL) recognized by the murine CD8 T cells and analyzed T cell activation by intracellular cytokine staining (ICS).

In the rMVA-HBc vaccination group we detected 5.9% core-specific CD8 T cells producing IFNγ, compared to 12.6% in the rMVA-HBc-CD70 vaccination group (*p* = 0.0086; Figure 2D,E). This confirmed that CD70 co-expression during boost vaccination fosters a functional CD8 T cell response. Of note, the results were confirmed by re-stimulation with an HBV core peptide pool at the individual mouse level (data not shown). A total of 2.6% (vaccination group rMVA-HBc) and 3.6% (vaccination group rMVA-HBc-CD70) of splenic CD8 T cells responded with IFNγ production to ex vivo stimulation with the dominant CD8 T cell epitope of the MVA-vector backbone B8R (Figure 2E). In this experiment, CD70 co-expression during boost vaccination resulted in a 2.1-fold increase in mean HBV core-specific CD8 T cell responses (corresponding to a difference of 1.81 standard deviations between both vaccination groups) and no significant difference in B8R-specific CD8 T cell responses (difference 1.01-fold standard deviations). Thus, the co-stimulatory effect of CD70 during boost vaccination seemed to mainly support HBV core-specific CD8 T cell responses and only had a minor effect on the un-primed MVA-specific CD8 T cell response. Of note also, no significant difference in terms of IFNγ production of CD4 T cells after re-stimulation with an HBV core peptide pool was observed between the vaccination groups (data not shown).

### 3.3. Transgene-Specific CD8 T Cells Gain Functionality by Co-Expression with CD70

Next, we used ICS to examine the ability of CD8 T cells to express multiple cytokines simultaneously as a measure of the quality of the HBV core-specific CD8 T cell response and found that the proportion of multifunctional CD8 T cells with respect to the expression of IFNγ, IL2 and TNF was increased in the HBc-CD70 group compared to the HBc-group 14 days after boost vaccination (Figure 2F). In order to investigate whether the proportion of C_93_-specific CD8 T cells producing IFNγ increased after CD70 co-expression, the percentage of IFNγ-producing CD8 T cells detected by ICS in relation to C_93_ multimer-positive CD8 T cells was determined for all mice individually. We detected a significant difference between the vaccination groups rMVA-HBc and rMVA-HBc-CD70 (*p* = 0.0129, Figure 2G) indicating the increased functionality of C_93_-specific CD8 T cells. CD70 co-expression activated about 20% of previously non-reactive C_93_-specific CD8 T cells in our setting, indicating that CD70 efficiently antagonizes tolerogenic signaling during CD8 T cell boosting. Taken together, CD70 co-expression improved both the quality and quantity of vaccine-induced, HBc-specific CD8 T cells in C57Bl/6 mice.

### 3.4. CD70 Co-Expression during Boost Vaccination Does Not Affect Humoral Immunity in a Therapeutic Vaccination Setting

Next, we evaluated the effect of CD70 co-expression during boost vaccination in a therapeutic vaccination approach using the *TherVacB* regime in HBV1.3.32 transgenic (HBV1.3tg) mice, which is a well-established animal model for the simulation of a chronic HBV infection in mice [51].

Previous studies have demonstrated that therapeutic efficacy of *TherVacB* vaccination depends on the antigen load in the HBV1.3tg mouse model [25]. Therefore, we used both low-intermediate and high viremic HBV1.3tg mice to evaluate the co-stimulatory effect of CD70 depending on the viral load. Hereby, hepatitis B e antigen (HBeAg) levels prior to treatment were determined utilizing the Abbott Architect HBeAg 2.0 assay to classify the treated mice discriminating between mice with low/intermediate (HBeAg 1-8 S/CO) and high viremia (HBeAg levels > 8 S/CO).

**Figure 3:**
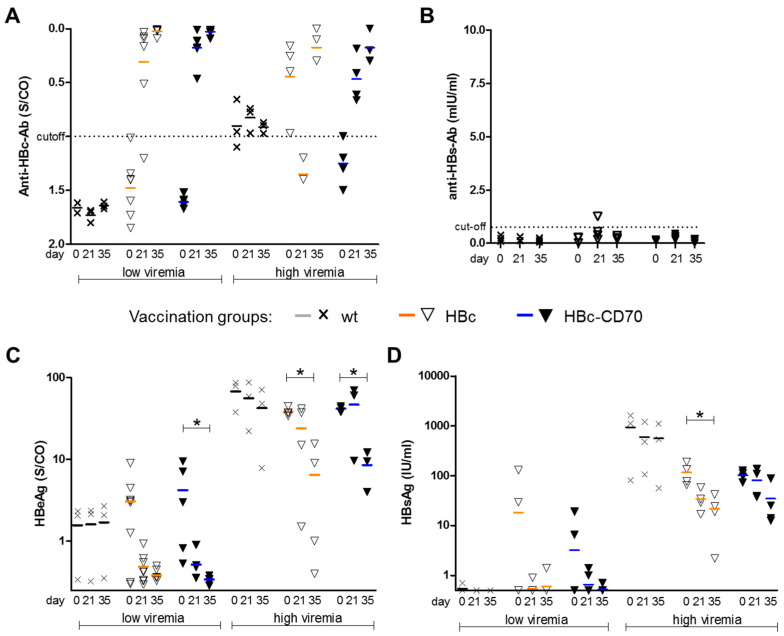
Development of HBV-specific antibodies and reduction in serum HBV antigens in HBV1.3tg mice receiving a protein-prime/MVA-boost vaccination. Serological markers were determined prior to the protein prime (day 0), after two protein vaccinations prior to the MVA boost (day 21) and seven days after the MVA boost (day 35). (**A**) Prior to vaccination, the mice were divided into groups that had either low/intermediate HBeAg levels (1-8 S/CO) or high antigen levels (>8 S/CO). (**A**,**B**) HBcAg-specific antibodies (anti-HBc) in these two groups of mice (**A**) and HBsAg-specific antibodies (anti-HBs) determined in low-viremic mice. (**C**,**D**) Serum HBeAg (**C**) and HBsAg (**D**) levels in low and high-viremic vaccinated mice. Crosses and triangles represent individual mice; Horizontal lines indicate mean values; Error bars indicate standard deviation. Statistics in (**A**) (**C**) and (**D**) were performed with one-way ANOVA, * *p*-value < 0.05.

First, we evaluated the effects of CD70 co-stimulation on the induction of humoral immunity. As expected, we observed that antibodies against HBc evolved in both vaccination groups (HBc and HBc-CD70) already after the protein-prime vaccination, and the MVA boost vaccination had no significant additional effect (Figure 3A). Further, no significant difference in the induction of HBc-specific antibodies was detectable between the two vaccination groups, indicating that there was no beneficial effect of CD70 in the production of anti-HBc. In addition, none of the vaccination regimes elicited a significant anti-HBs response, as expected (Figure 3B). Our results therefore do not indicate that CD70 co-stimulation affected humoral immunity in our therapeutic vaccination setting.

### 3.5. CD70 Co-Expression during Boost Vaccination Has No Influence on HBeAg and HBsAg Levels

Next, we examined the effect of CD70 co-expression during boost vaccination on serum HBeAg and HBsAg levels, which serve as markers for HBV replication. In most vaccinated mice (from both groups, HBc and HBc-CD70), HBeAg levels decreased to low or undetectable levels after boost vaccinations in mice with low viremia (Figure 3C). In high viremic mice the levels of HBeAg significantly decreased after immunization. Assessment of the vaccination effects on the serum HBsAg was only possible in the high-viremic mice, since most of the mice with low viremia demonstrated undetectable HBsAg at the beginning of the experiments (Figure 3D), confirming our previous reports [25]. In contrast to controls (wt), vaccination of high-viremic mice with HBc and HBc-CD70 regimens resulted in an approximately 1-log_10_ drop in HBsAg levels at day 35 as compared to the baseline values. However, no additional effect of CD70 co-expression on lowering neither HBeAg, nor HBsAg levels was observed.

### 3.6. CD70 Co-Expression during Boost Vaccination Enhances the Number of Transgene-Specific CD8 T Cells in a Therapeutic Vaccination Setting Using rMVA-HBc-CD70

**Figure 4:**
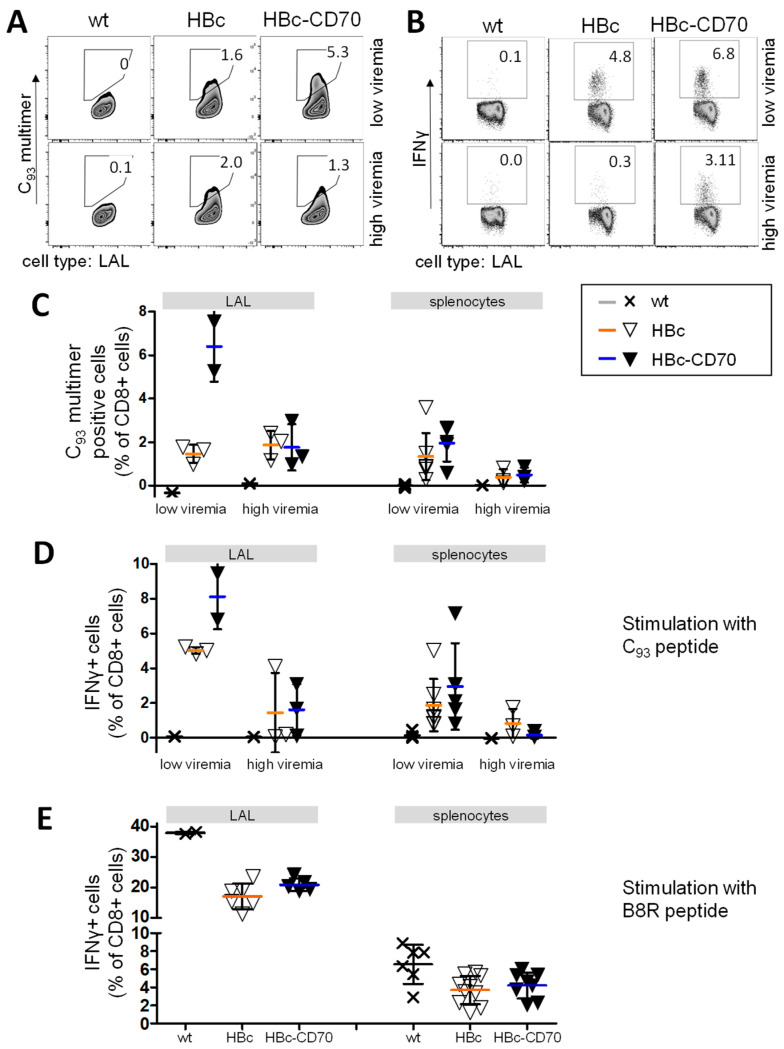
Amount and functionality of CD8 T cells in the livers and spleens of vaccinated HBV1.3tg mice with and without CD70 co-expression. HBV1.3tg mice were immunized with protein-prime (day 0, HBc + CpG + PCEP, group wt only CpG and PCEP) and MVA boost (day 21, with MVAwt (group wt), rMVA-HBc (group HBc) or rMVA-HBc-CD70 (group HBc-CD70)). Mice were divided into groups with low to moderate HBeAg levels (1–8 S/CO, “low viremia”) and high antigen levels (8–23 S/CO, “high viremia”) before the experiment. At day 35, splenocytes and liver-associated lymphocytes of mice were isolated. (**A**) Representative dot blot analysis of liver-associated lymphocytes (LAL) for single mice of each vaccination group, utilizing C_93_ multimer staining. (**B**) Representative dot blot analysis of intracellular cytokine staining (IFNγ) after ex vivo C_93_ stimulation of LALs. (**C**) Percentages of C_93_ multimer-positive CD8 T cells in vaccinated mice. (**D**) HBV core-specific CD8 T cell response for the groups of mice with low and high viremia represented by the portion of CD8 T cells expressing IFNγ after ex vivo stimulation with C_93_ peptide. (**E**) MVA-specific CD8 T cell response represented by the portion of CD8 T cells expressing IFNγ after ex vivo stimulation with B8R peptide. Mice with low and high viremia are presented together. C_93_: HBV core-derived CD8 T cell epitope; B8R: MVA-derived CD8 T cell epitope; Crosses and triangles represent individual mice; Horizontal lines indicate mean values; Error bars indicate standard deviation. Differences between groups HBc and HBc-CD70 (if >2 mice/group) were not statistically significant (one-way ANOVA and unpaired t-test).

We next analyzed the number of HBV core-specific CD8 T cells derived from the spleens and livers of vaccinated HBV1.3tg mice by C_93_ multimer staining (Figure 4). As expected, an inverse correlation of viremia and HBV core-specific CD8 T cell responses was observed in our vaccination experiments (Figure 4A,C) corresponding to earlier results [25].

The functionality of C_93_-specific CD8 T cells was assessed by ICS (Figure 4B,D). In low viremic mice, CD70 co-expression during boost vaccination resulted in a slightly higher proportion of IFNγ-producing CD8 T cells in the liver and spleen (not significant), comparable to our results in antigen-naïve C75Bl/6 mice. In high viremic mice, however, no increase in HBV core-specific T cell immunity was detected and CD70 co-expression may even have had an unwanted effect such as a reduced IFNγ production in the spleen (see Figure 4D, splenocytes).

Examination of the vector-specific immune response by intracellular cytokine staining after stimulation with the MVA-derived peptide B8R (Figure 4E) showed that CD70 co-expression also had no noticeable effect on the strength of the vector-specific CD8 T cell response.

In summary, CD70-costimulation increased the number of HBV core-specific CD8 T cells as well as the number of IFNγ-producing core-specific T cells upon vaccination in low-medium viremic mice. However, HBV core-specific immune tolerance could not be overcome in high viremic, HBV-transgenic mice by CD70 co-expression.

### 3.7. Co-Expression by CD70 during Boost Vaccination Increases the Number of T Cell Clusters in Livers of Vaccinated HBV1.3tg Mice without Promoting Immunopathology

**Figure 5:**
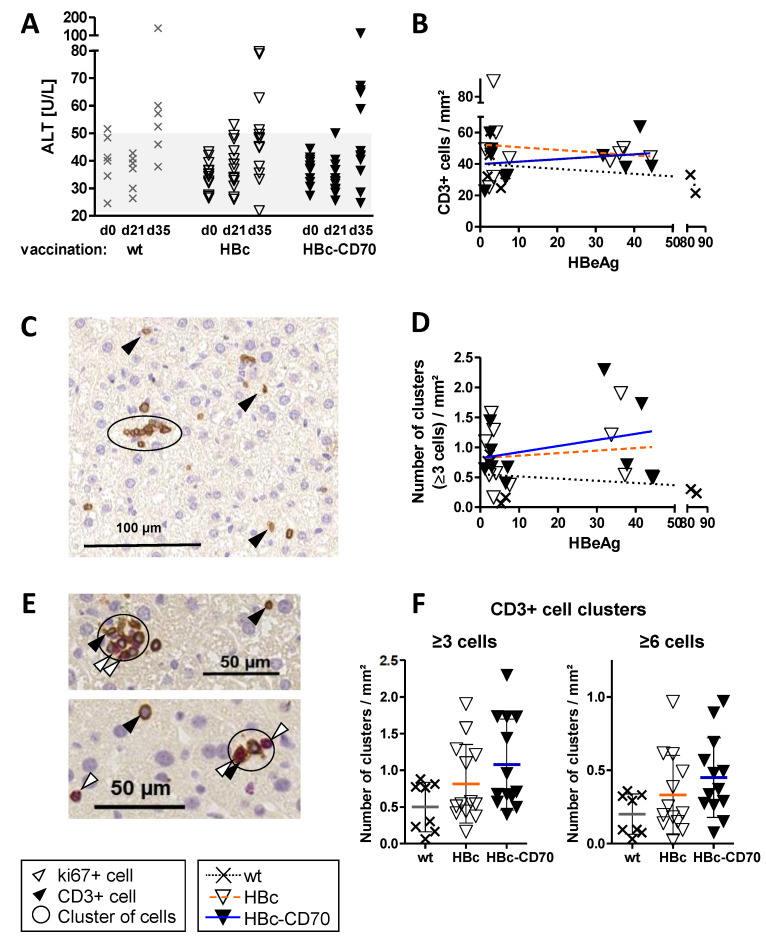
Serum alanine transaminase (ALT) levels and T cell infiltration into livers of HBV1.3tg mice after therapeutic vaccination with rMVA-HBc ± CD70. HBV1.3tg mice were immunized subcutaneously with 30 µg of HBcAg adjuvanted with 32 µg CpG and 25 µg PCEP at day 0. At day 21, mice received 10^8^ IU rMVA-HBc (group HBc) or rMVA-HBc-CD70 (group HBc-CD70) by intraperitoneal injection. A control group received adjuvants only without the addition of protein and 10^8^ IU MVAwt (group wt). At day 35, mice were sacrificed and liver sections were stained for CD3+ (T cells) and Ki67+ cells (dividing cells). (**A**) Serum ALT levels were measured at day 0, 21 and 35. Light grey: normal range of ALT levels in mice. (**B**) In liver sections that were stained for CD3, CD3+ cells were counted in multiple sections. Data are shown in correlation to the HBeAg-levels (S/CO) that complied with the viremia of mice at day 0. (**C**) Representative liver section after staining for CD3. (**D**) The entire section was scanned for multiple CD3+ cells in direct cell contact (=cell clusters). Clusters of at least 3 cells in direct contact were counted and set in relation to the surface of the section. Data is shown in relation to the HBeAg level (S/CO). (**E**) Representative pictures of liver sections that were stained with anti CD3 and anti Ki67 antibodies showing double positive cells as part of a cell cluster. (**F**) In addition to clusters of more than 3 cells in direct contact (**D**), also clusters of at least 6 cells in direct contact were counted in the same way. Data are presented irrespective of the HBeAg level. Data in (**F**) were not statistically relevant in a one-way ANOVA. Crosses and triangles represent individual mice; horizontal lines indicate mean values; error bars indicate standard deviation.

To investigate whether CD70 co-expression may promote liver damage, we measured serum alanine transaminase (ALT) activity in serum. In some of the vaccinated HBV1.3tg mice we found a minor ALT increase independent of the vaccination group at day 35 (Figure 5A). This was most probably caused by the MVA boost vaccination per se. In the HBc-CD70 group, no elevated ALT-levels in general or increased numbers of mice with high ALT-levels (>100 U/L) were detected.

Finally, we investigated the infiltration of T cells into the liver in the therapeutic vaccination setting. By immunohistochemistry staining for CD3 in liver biopsies we observed similar absolute numbers of T cells in HBV1.3tg mice in all vaccination groups (wt, HBc and HBc-CD70). The number of T cells showed no correlation with the viremia of mice (Figure 5B). The number of T cell clusters (≥3 or ≥6 cells in direct contact) appeared to be increased in vaccination groups HBc and HBc-CD70 but did also not correlate with the viremia of mice (Figure 5D). In a double staining of liver sections (CD3 for T cells and Ki67 for proliferating cells) we noticed that T cells in cell clusters frequently expressed Ki67 (Figure 5E). As both the number of single T cells and T cells in clusters did not correlate with changes of viremia in the HBV1.3tg mice, the statistical analysis of T cell clusters in liver sections was performed on all mice together (Figure 5F); compared to the control group (wt), the number of T cell clusters was increased in both vaccination groups HBc and HBc-CD70 to 0.33 and 0.45 clusters/mm^2^, respectively, compared to 0.2 clusters/mm^2^ in the control group (wt) (Figure 5E, clusters of ≥ 6 cells). The improvement was highest in the vaccination group HBc-CD70. Taken together, these results suggest that therapeutic vaccination using a protein-prime, MVA-vector boost scheme leads to T cell proliferation in the liver, which may be enhanced by the co-expression of CD70.

## 4. Discussion

In this study, we investigated the potential of co-expressing CD70 in the context of an MVA-vector vaccine to enhance the quantity and quality of the CD8 T cell response directed against a transgenic target utilizing a protein-prime, MVA-boost vaccination strategy [25,26]. We therefore integrated a CD70 coding sequence into an MVA-vector under the same promotor as an HBV core open reading frame serving as a model antigen and showed the co-expression of HBV core protein and CD70. In HBV-naïve mice, co-expression of CD70 during boost vaccination doubled the number of HBV core-specific CD8 T cells and improved their functionality indicated by the secretion of IFNγ, TNFα and IL2, while T cell immunity specific for the MVA-vector was not significantly affected. Furthermore, CD70 co-expression was able to trigger IFNγ production in a higher portion of HBV core-specific CD8 T cells after vaccination in comparison to mice receiving vaccination without CD70 co-expression resulting in increased levels of functional T cells. In HBV1.3tg mice, a minor effect was observed on T cells derived from the spleen and liver of low-viremic mice after vaccination. In high viremic mice, however, the immune response after vaccination was reduced independently of the co-expression of CD70. Additionally, no benefit of CD70 co-expression could be detected.

Liver histology of the HBV1.3tg mice showed that CD70 co-expression increased the number of vaccine-induced, proliferative T cell clusters. We thus conclude that T cells in proliferative cell clusters are most likely HBV core-specific and that CD70 co-expression has a beneficial effect on T cell proliferation. The overall number of CD3+ cells in the liver was not influenced by vaccination. This was expected as the majority are single T cells that traffic through the liver but are not specific for HBV core [16]. Interestingly, the number of T cell clusters was independent of the viremia of mice while the analysis of HBV core-specific T cells by flow cytometry showed the previously observed negative correlation (lower CD8 T cell responses in high viremic mice). In conclusion, in low-medium viremic mice, CD70 co-expression during boost vaccination seems to provide benefits for T cell function and proliferation, especially in the liver, while in high viremic mice, these effects could not be confirmed. A high antigen load is assumed to be one factor which leads to immune tolerance enabling chronic infection and is one of the main hurdles in preclinical studies of therapeutic vaccination against chronic HBV infection [25,26]. At this place, co-expression with CD70 was not able to overcome this obstacle and it has been shown that other strategies such as the knockdown of antigens prior to the therapeutic vaccination are more promising [26].

The current study is limited by the fact that only one antigen was used and only one prime and one boost vaccination was applied in contrast to more elaborate vaccination schemes such as the *TherVacB* scheme [26]. Whether the potential of CD70 co-expression in a therapeutic vaccination setting against hepatitis B is underestimated by the choice of the HBV core instead of the more potent HBV surface antigen as a model was not assessed and would be an interesting subject for further investigation. An additional limitation was the model used because HBV1.3tg mice have a depleted core-specific T cell repertoire because they start expressing the HBV core antigen already before birth. This limits the predictive value of this study for a therapeutic hepatitis B vaccine.

However, our data provide valuable information on how CD70 can be used as a safe component in a boost-vector design to precisely enhance transgene-specific CD8 responses to a vaccine antigen. In a physiological setting, CD70 expression on immune cells is highly regulated [39] and overexpression and prolonged exposure to CD70 can have detrimental effects on cellular immunity leading to exhaustion of the naïve T cell pool [52]. Hence, it was important to show that delivery by MVA is a safe application of this co-stimulator: infected cells undergo apoptosis within a few days [53], but this time-frame seemed to be sufficient to selectively improve the desired CD8 T cell recall response. There is evidence that MVA preferentially infects antigen-presenting dendritic cells, which upon infection with MVA are impaired in their maturation due to the shutdown of cellular protein production in MVA infection [53,54,55]. CD70 is naturally upregulated in mature dendritic cells and helps to form the effector and memory T cell pool [30,39]. Transgenic CD70 provided by rMVA could compensate this the lack of maturation and cause the observed improvements in CD8 T cell effector function. It has been shown that the expression of CD70 by immature dendritic cells is capable of converting CD8 T cells from a tolerogenic to an immunogenic state which interestingly occurs independently of CD4 T cell help [56,57].

Bathke et al. [58] designed rMVA-CD70 constructs for homologous prime-boost experiments and observed that CD8 T cell responses against the MVA epitope B8R were stronger after CD70 co-expression via two rMVA immunizations than those against the target transgene—ovalbumin (OVA). Such misdirected effects could lead to unspecific and harmful immune reactions. With our vaccination scheme and vector design, we obtained a stronger improvement of CD8 T cell responses against HBV core as the model antigen than that against the MVA-specific epitope B8R. The major difference in the study design was our protein prime that raised the target-specific T cell responses over those directed against that of the viral backbone [25]. At the time of boost immunization, HBV core-specific CD8 T cells were already present in our mice. The boost itself was described to shape the immunodominance hierarchy [59] and appeared in our experiments even to be significantly improvable by CD70 co-expression—at least in naive mice. However, MVA-specific T cells were not yet present in our mice at the time of boost, so the MVA antigens in our experiments represented a baseline immunization that has already been shown to play no role in antigen competition [59]. Further, Bathke et al. expressed CD70 under the pHyb promoter while the target antigen OVA was expressed under the pS promoter. We spatially and temporally coupled the transgene expression of CD70 and the target gene utilizing only one promoter mH5 to generate one type of transcript, which via the P2A site results in equimolar expression of CD70 protein and the vaccine antigen. We consider this essential to drive the co-stimulation of cellular immunity against the target antigen.

In summary, we present here a safe system to selectively enhance the vector-vaccine-induced CD8 T cell response to the target antigen through CD70 co-expression during boost immunization. In this respect, our study may contribute to the development and improvement of therapeutic vaccines revealing novel generic design principles and thus foster advances of current strategies to fight malignancies and persistent viral infections.

## Data Availability

Not applicable.

## References

[1] Collaborators P.O. (2018). Global prevalence, treatment, and prevention of hepatitis B virus infection in 2016: A modelling study. Lancet Gastroenterol. Hepatol..

[2] WHO (2021). Global Progress Report on HIV, Viral Hepatitis and Sexually Transmitted Infections. https://www.who.int/publications/i/item/9789240027077.

[3] EASL (2017). EASL 2017 Clinical Practice Guidelines on the management of hepatitis B virus infection. J. Hepatol..

[4] Cornberg M., Sandmann L., Protzer U., Niederau C., Tacke F., Berg T., Glebe D., Jilg W., Wedemeyer H., Wirth S. (2021). S3-Leitlinie der Deutschen Gesellschaft für Gastroenterologie, Verdauungs- und Stoffwechselkrankheiten (DGVS) zur Prophylaxe, Diagnostik und Therapie der Hepatitis-B-Virusinfektion–(AWMF-Register-Nr. 021-11). Z. Fur Gastroenterol..

[5] Lok A.S., Zoulim F., Dusheiko G., Ghany M.G. (2017). Hepatitis B cure: From discovery to regulatory approval. J. Hepatol..

[6] Terrault N.A., Bzowej N.H., Chang K.M., Hwang J.P., Jonas M.M., Murad M.H. (2016). AASLD guidelines for treatment of chronic hepatitis B. Hepatology.

[7] Chisari F.V., Ferrari C. (1995). Hepatitis B virus immunopathogenesis. Annu. Rev. Immunol..

[8] Chisari F.V., Isogawa M., Wieland S.F. (2010). Pathogenesis of hepatitis B virus infection. Pathol. -Biol..

[9] Hoogeveen R.C., Robidoux M.P., Schwarz T., Heydmann L., Cheney J.A., Kvistad D., Aneja J., Melgaço J.G., Fernandes C.A., Chung R.T. (2019). Phenotype and function of HBV-specific T cells is determined by the targeted epitope in addition to the stage of infection. Gut.

[10] Maini M.K., Boni C., Ogg G.S., King A.S., Reignat S., Lee C.K., Larrubia J.R., Webster G.J., McMichael A.J., Ferrari C. (1999). Direct ex vivo analysis of hepatitis B virus-specific CD8(+) T cells associated with the control of infection. Gastroenterology.

[11] Thimme R., Wieland S., Steiger C., Ghrayeb J., Reimann K.A., Purcell R.H., Chisari F.V. (2003). CD8(+) T cells mediate viral clearance and disease pathogenesis during acute hepatitis B virus infection. J. Virol..

[12] Boni C., Fisicaro P., Valdatta C., Amadei B., Di Vincenzo P., Giuberti T., Laccabue D., Zerbini A., Cavalli A., Missale G. (2007). Characterization of hepatitis B virus (HBV)-specific T-cell dysfunction in chronic HBV infection. J. Virol..

[13] Chang J.J., Thompson A.J., Visvanathan K., Kent S.J., Cameron P.U., Wightman F., Desmond P., Locarnini S.A., Lewin S.R. (2007). The phenotype of hepatitis B virus-specific T cells differ in the liver and blood in chronic hepatitis B virus infection. Hepatology.

[14] Das A., Hoare M., Davies N., Lopes A.R., Dunn C., Kennedy P.T., Alexander G., Finney H., Lawson A., Plunkett F.J. (2008). Functional skewing of the global CD8 T cell population in chronic hepatitis B virus infection. J. Exp. Med..

[15] Klenerman P., Hill A. (2005). T cells and viral persistence: Lessons from diverse infections. Nat. Immunol..

[16] Maini M.K., Boni C., Lee C.K., Larrubia J.R., Reignat S., Ogg G.S., King A.S., Herberg J., Gilson R., Alisa A. (2000). The role of virus-specific CD8(+) cells in liver damage and viral control during persistent hepatitis B virus infection. J. Exp. Med..

[17] Tan A.T., Loggi E., Boni C., Chia A., Gehring A.J., Sastry K.S., Goh V., Fisicaro P., Andreone P., Brander C. (2008). Host ethnicity and virus genotype shape the hepatitis B virus-specific T-cell repertoire. J. Virol..

[18] Webster G.J., Reignat S., Brown D., Ogg G.S., Jones L., Seneviratne S.L., Williams R., Dusheiko G., Bertoletti A. (2004). Longitudinal analysis of CD8+ T cells specific for structural and nonstructural hepatitis B virus proteins in patients with chronic hepatitis B: Implications for immunotherapy. J. Virol..

[19] Barnes E. (2015). Therapeutic vaccines in HBV: Lessons from HCV. Med. Microbiol. Immunol..

[20] Cargill T., Barnes E. (2021). Therapeutic vaccination for treatment of chronic hepatitis B. Clin. Exp. Immunol..

[21] Dembek C., Protzer U., Roggendorf M. (2018). Overcoming immune tolerance in chronic hepatitis B by therapeutic vaccination. Curr. Opin. Virol..

[22] Liu J., Kosinska A., Lu M., Roggendorf M. (2014). New therapeutic vaccination strategies for the treatment of chronic hepatitis B. Virol. Sin..

[23] Kosinska A.D., Liu J., Lu M., Roggendorf M. (2015). Therapeutic vaccination and immunomodulation in the treatment of chronic hepatitis B: Preclinical studies in the woodchuck. Med. Microbiol. Immunol..

[24] Schurich A., Khanna P., Lopes A.R., Han K.J., Peppa D., Micco L., Nebbia G., Kennedy P.T., Geretti A.M., Dusheiko G. (2011). Role of the coinhibitory receptor cytotoxic T lymphocyte antigen-4 on apoptosis-Prone CD8 T cells in persistent hepatitis B virus infection. Hepatology.

[25] Backes S., Jäger C., Dembek C.J., Kosinska A.D., Bauer T., Stephan A.S., Dišlers A., Mutwiri G., Busch D.H., Babiuk L.A. (2016). Protein-prime/modified vaccinia virus Ankara vector-boost vaccination overcomes tolerance in high-antigenemic HBV-transgenic mice. Vaccine.

[26] Michler T., Kosinska A.D., Festag J., Bunse T., Su J., Ringelhan M., Imhof H., Grimm D., Steiger K., Mogler C. (2020). Knockdown of Virus Antigen Expression Increases Therapeutic Vaccine Efficacy in High-Titer Hepatitis B Virus Carrier Mice. Gastroenterology.

[27] Bunse T., Kosinska A.D., Michler T., Protzer U. (2022). PD-L1 Silencing in Liver Using siRNAs Enhances Efficacy of Therapeutic Vaccination for Chronic Hepatitis B. Biomolecules.

[28] Kosinska A.D., Moeed A., Kallin N., Festag J., Su J., Steiger K., Michel M.L., Protzer U., Knolle P.A. (2019). Synergy of therapeutic heterologous prime-boost hepatitis B vaccination with CpG-application to improve immune control of persistent HBV infection. Sci. Rep..

[29] Hintzen R.Q., Lens S.M., Lammers K., Kuiper H., Beckmann M.P., van Lier R.A. (1995). Engagement of CD27 with its ligand CD70 provides a second signal for T cell activation. J. Immunol..

[30] Arens R., Tesselaar K., Baars P.A., van Schijndel G.M., Hendriks J., Pals S.T., Krimpenfort P., Borst J., van Oers M.H., van Lier R.A. (2001). Constitutive CD27/CD70 interaction induces expansion of effector-type T cells and results in IFNgamma-mediated B cell depletion. Immunity.

[31] Hendriks J., Gravestein L.A., Tesselaar K., van Lier R.A., Schumacher T.N., Borst J. (2000). CD27 is required for generation and long-term maintenance of T cell immunity. Nat. Immunol..

[32] Hendriks J., Xiao Y., Borst J. (2003). CD27 promotes survival of activated T cells and complements CD28 in generation and establishment of the effector T cell pool. J. Exp. Med..

[33] Yamada A., Salama A.D., Sho M., Najafian N., Ito T., Forman J.P., Kewalramani R., Sandner S., Harada H., Clarkson M.R. (2005). CD70 signaling is critical for CD28-independent CD8+ T cell-mediated alloimmune responses in vivo. J. Immunol..

[34] Boursalian T.E., McEarchern J.A., Law C.L., Grewal I.S. (2009). Targeting CD70 for human therapeutic use. Adv. Exp. Med. Biol..

[35] Roberts D.J., Franklin N.A., Kingeter L.M., Yagita H., Tutt A.L., Glennie M.J., Bullock T.N. (2010). Control of established melanoma by CD27 stimulation is associated with enhanced effector function and persistence, and reduced PD-1 expression of tumor infiltrating CD8(+) T cells. J. Immunother..

[36] Soares H., Waechter H., Glaichenhaus N., Mougneau E., Yagita H., Mizenina O., Dudziak D., Nussenzweig M.C., Steinman R.M. (2007). A subset of dendritic cells induces CD4+ T cells to produce IFN-gamma by an IL-12-independent but CD70-dependent mechanism in vivo. J. Exp. Med..

[37] Denoeud J., Moser M. (2011). Role of CD27/CD70 pathway of activation in immunity and tolerance. J. Leukoc. Biol..

[38] Glouchkova L., Ackermann B., Zibert A., Meisel R., Siepermann M., Janka-Schaub G.E., Goebel U., Troeger A., Dilloo D. (2009). The CD70/CD27 pathway is critical for stimulation of an effective cytotoxic T cell response against B cell precursor acute lymphoblastic leukemia. J. Immunol..

[39] Schildknecht A., Miescher I., Yagita H., van den Broek M. (2007). Priming of CD8+ T cell responses by pathogens typically depends on CD70-mediated interactions with dendritic cells. Eur. J. Immunol..

[40] Xiao Y., Peperzak V., Keller A.M., Borst J. (2008). CD27 instructs CD4+ T cells to provide help for the memory CD8+ T cell response after protein immunization. J. Immunol..

[41] Jacobs J., Deschoolmeester V., Zwaenepoel K., Rolfo C., Silence K., Rottey S., Lardon F., Smits E., Pauwels P. (2015). CD70: An emerging target in cancer immunotherapy. Pharmacol. Ther..

[42] Arroyo Hornero R., Georgiadis C., Hua P., Trzupek D., He L.Z., Qasim W., Todd J.A., Ferreira R.C., Wood K.J., Issa F. (2020). CD70 expression determines the therapeutic efficacy of expanded human regulatory T cells. Commun. Biol..

[43] Hodi F.S., O’Day S.J., McDermott D.F., Weber R.W., Sosman J.A., Haanen J.B., Gonzalez R., Robert C., Schadendorf D., Hassel J.C. (2010). Improved survival with ipilimumab in patients with metastatic melanoma. N. Engl. J. Med..

[44] van de Ven K., Borst J. (2015). Targeting the T-cell co-stimulatory CD27/CD70 pathway in cancer immunotherapy: Rationale and potential. Immunotherapy.

[45] Kosinska A.D., Festag J., Mück-Häusl M., Festag M.M., Asen T., Protzer U. (2021). Immunogenicity and Antiviral Response of Therapeutic Hepatitis B Vaccination in a Mouse Model of HBeAg-Negative, Persistent HBV Infection. Vaccines.

[46] Wyatt L.S., Earl P.L., Xiao W., Americo J.L., Cotter C.A., Vogt J., Moss B. (2009). Elucidating and minimizing the loss by recombinant vaccinia virus of human immunodeficiency virus gene expression resulting from spontaneous mutations and positive selection. J. Virol..

[47] Sino Biologocal Inc. Human CD70/CD27L/TNFSF7 Protein (Fc Tag): Datasheet, Catalog Number 10780-H01H. Human CD70 Datasheet..

[48] Stross L., Günther J., Gasteiger G., Asen T., Graf S., Aichler M., Esposito I., Busch D.H., Knolle P., Sparwasser T. (2012). Foxp3+ regulatory T cells protect the liver from immune damage and compromise virus control during acute experimental hepatitis B virus infection in mice. Hepatology.

[49] Luke G.A., de Felipe P., Lukashev A., Kallioinen S.E., Bruno E.A., Ryan M.D. (2008). Occurrence, function and evolutionary origins of ‘2A-like’ sequences in virus genomes. J. Gen. Virol..

[50] Wang Z., Martinez J., Zhou W., La Rosa C., Srivastava T., Dasgupta A., Rawal R., Li Z., Britt W.J., Diamond D. (2010). Modified H5 promoter improves stability of insert genes while maintaining immunogenicity during extended passage of genetically engineered MVA vaccines. Vaccine.

[51] Guidotti L.G., Matzke B., Schaller H., Chisari F.V. (1995). High-level hepatitis B virus replication in transgenic mice. J. Virol..

[52] Tesselaar K., Arens R., van Schijndel G.M., Baars P.A., van der Valk M.A., Borst J., van Oers M.H., van Lier R.A. (2003). Lethal T cell immunodeficiency induced by chronic costimulation via CD27-CD70 interactions. Nat. Immunol..

[53] Chahroudi A., Garber D.A., Reeves P., Liu L., Kalman D., Feinberg M.B. (2006). Differences and similarities in viral life cycle progression and host cell physiology after infection of human dendritic cells with modified vaccinia virus Ankara and vaccinia virus. J. Virol..

[54] Altenburg A.F., van de Sandt C.E., Li B.W.S., MacLoughlin R.J., Fouchier R.A.M., van Amerongen G., Volz A., Hendriks R.W., de Swart R.L., Sutter G. (2017). Modified Vaccinia Virus Ankara Preferentially Targets Antigen Presenting Cells In Vitro, Ex Vivo and In Vivo. Sci. Rep..

[55] Engelmayer J., Larsson M., Subklewe M., Chahroudi A., Cox W.I., Steinman R.M., Bhardwaj N. (1999). Vaccinia virus inhibits the maturation of human dendritic cells: A novel mechanism of immune evasion. J. Immunol..

[56] Bullock T.N., Yagita H. (2005). Induction of CD70 on dendritic cells through CD40 or TLR stimulation contributes to the development of CD8+ T cell responses in the absence of CD4+ T cells. J. Immunol..

[57] Keller A.M., Xiao Y., Peperzak V., Naik S.H., Borst J. (2009). Costimulatory ligand CD70 allows induction of CD8+ T-cell immunity by immature dendritic cells in a vaccination setting. Blood.

[58] Bathke B., Pätzold J., Kassub R., Giessel R., Lämmermann K., Hinterberger M., Brinkmann K., Chaplin P., Suter M., Hochrein H. (2018). CD70 encoded by modified vaccinia virus Ankara enhances CD8 T-cell-dependent protective immunity in MHC class II-deficient mice. Immunology.

[59] Kastenmuller W., Gasteiger G., Gronau J.H., Baier R., Ljapoci R., Busch D.H., Drexler I. (2007). Cross-competition of CD8+ T cells shapes the immunodominance hierarchy during boost vaccination. J. Exp. Med..

